# Hormonal Contraception Is Associated with a Reduced Risk of Bacterial Vaginosis: A Systematic Review and Meta-Analysis

**DOI:** 10.1371/journal.pone.0073055

**Published:** 2013-09-04

**Authors:** Lenka A. Vodstrcil, Jane S. Hocking, Matthew Law, Sandra Walker, Sepehr N. Tabrizi, Christopher K. Fairley, Catriona S. Bradshaw

**Affiliations:** 1 Sexual Health Unit, Melbourne School of Population and Global Health, University of Melbourne, Parkville, Australia; 2 Melbourne Sexual Health Centre, Alfred Hospital, Carlton, Australia; 3 Murdoch Children’s Research Institute, Parkville, Australia; 4 Centre for Women’s Health, Gender and Society, Melbourne School of Population and Global Health, University of Melbourne, Parkville, Australia; 5 Kirby Institute, University of New South Wales, Coogee, Australia; 6 Department of Microbiology and Infectious Diseases, The Royal Women’s Hospital, Parkville, Victoria, Australia; 7 Department of Obstetrics and Gynaecology, University of Melbourne, The Royal Women’s Hospital Parkville, Victoria, Australia; 8 Department of Epidemiology and Preventative Medicine, Monash University, Melbourne, Australia; University of Ottawa, Canada

## Abstract

**Objective:**

To examine the association between hormonal contraception (HC) and bacterial vaginosis (BV) by systematic review and meta-analysis.

**Methods:**

Medline, Web of Science and Embase databases were searched to 24/1/13 and duplicate references removed. Inclusion criteria 1) >20 BV cases; 2) accepted BV diagnostic method; 3) measure of HC-use either as combined oestrogen-progesterone HC (combined), progesterone-only contraception (POC) or unspecified HC (u-HC); 4) ≥10% of women using HC; 5) analysis of the association between BV and HC-use presented; 6) appropriate control group. Data extracted included: type of HC, BV diagnostic method and outcome (prevalent, incident, recurrent), and geographical and clinic-setting. Meta-analyses were conducted to calculate pooled effect sizes (ES), stratified by HC-type and BV outcome. This systematic review is registered with PROSPERO (CRD42013003699).

**Results:**

Of 1713 unique references identified, 502 full-text articles were assessed for eligibility and 55 studies met inclusion criteria. Hormonal contraceptive use was associated with a significant reduction in the odds of prevalent BV (pooled effect size by random-effects [reES] = 0.68, 95%CI0.63–0.73), and in the relative risk (RR) of incident (reES = 0.82, 95%CI:0.72–0.92), and recurrent (reES = 0.69, 95%CI:0.59–0.91) BV. When stratified by HC-type, combined-HC and POC were both associated with decreased prevalence of BV and risk of incident BV. In the pooled analysis of the effect of HC-use on the composite outcome of prevalent/incident/recurrent BV, HC-use was associated with a reduced risk of any BV (reES = 0.78, 95%CI:0.74–0.82).

**Conclusion:**

HC-use was associated with a significantly reduced risk of BV. This negative association was robust and present regardless of HC-type and evident across all three BV outcome measures. When stratified by HC-type, combined-HC and POC were both individually associated with a reduction in the prevalence and incidence of BV. This meta-analysis provides compelling evidence that HC-use influences a woman’s risk of BV, with important implications for clinicians and researchers in the field.

## Introduction

Bacterial Vaginosis (BV) is the most common cause of vaginal discharge in reproductive age women, of unknown, but probable polymicrobial aetiology. BV is associated with significant clinical sequelae including increased risk of HIV acquisition [1], [2], preterm delivery [3], [4] and pelvic inflammatory disease (PID) [5]. While there have been limited population-based studies, the National Health and Nutrition Survey in the United States reported a BV prevalence in 17–49 year old women of 29% [6], and an Australian study in 17–25 year old women attending general and reproductive health services found a BV prevalence of 12% [7]. Treatment with current first line antibiotics have similar short-term efficacy with 70–80% cure rates at one month [8], [9], however BV recurrence is common, with rates as high as 58% within 12 months [10]. No sustained improvement in cure has been derived from combining first line antibiotic therapies, using suppressive antibiotic regimens or with adjunctive probiotic approaches [11].

Interestingly, a number of observational studies have reported that women using hormonal contraceptives have a reduced risk of prevalent [12]–[15] and recurrent [10], [16], [17] BV. These data particularly reflect the use of combined oestrogen-progesterone contraceptive agents or “unspecified hormonal contraceptives”, but several studies report a reduced risk of incident and recurrent BV in progesterone-only contraceptive users [16], [18]. With over 50% of women experiencing BV recurrence following first-line antibiotic therapies, and no significant improvement in the management of BV in the last 20 years, identifying potential modifiable practices that influence susceptibility to BV and recurrence are integral to progressing prevention and management approaches for this important and common genital tract condition. This systematic review and meta-analysis examines available data on the association between hormonal contraception (HC) use, specific types of HC-use and the outcomes of prevalent, incident and recurrent BV.

## Methods

We used the PRISMA statement to guide this systematic review and meta-analysis [19].

### Protocol and Registration

Analysis methods and inclusion criteria were specified in advance and documented in a protocol registered with prospective registration of systematic reviews (PROSPERO), registration number: CRD42013003699 (http://www.crd.york.ac.uk/PROSPERO/).

### Eligibility Criteria

#### Types of studies

We searched for all peer-reviewed, English language, studies published before January 2013 that reported an association between BV and HC. Conference abstracts identified in searches were reviewed. Review, editorial and discussion articles were excluded but reference lists were examined.

#### Types of participants, hormonal contraceptive use and outcome measures

Studies including women of any age investigated for prevalent, incident or recurrent BV, using an established published diagnostic method for BV, such as the Nugent, Amsel, Ison-Hay, Spiegel and modified Amsel methods, were assessed for eligibility. Cohort, cross-sectional and randomised controlled trials (RCTs) were considered for inclusion. Eligible studies had to include a measure of HC-use, exposure to HCs in ≥10% of the study population, and compare HC-users to women not using HC. HC-use included combined oestrogen-progesterone contraception (combined), progesterone-only contraception (POC) and use of an unspecified HC. Studies were ineligible if they were: animal studies, exclusively consisted of post-menopausal or pregnant women, used non-standard BV diagnostic methods, had <20 cases of BV, <10% of participants using HC, did not have a control/comparator group, or if the control group was exclusively users of intra-uterine devices (IUDs), which have been reported to increase the risk of BV. Studies in which there was no analysis of the association between BV and HC-use presented in the manuscript were excluded. In studies reporting >1 BV outcome measure, such as prevalent and incident BV, or >1 type of HC-type, each outcome and/or HC-type was separately included, if they were mutually exclusive of one another.

### Search Strategy

Studies were predominantly identified by searching electronic databases. Language was limited to English, and any non-English articles that were identified were excluded from the analysis. Our search was applied to the databases Medline (Web of Knowledge [Pubmed]), Web of Science (Web of Knowledge) and Embase (Ovid) until January 24^th^ 2013. We also reviewed reference lists of selected studies for other potentially relevant studies.

### Search

We used the following search terms to search all databases: ((bacterial vaginosis) OR (vaginosis) OR (bacterial infections and vaginitis) OR (gardnerella)) AND ((hormonal contraceptive) OR (hormone) OR (contraceptive) OR (contraception) OR (oestrogen) OR (progesterone) OR (progestin) OR (Intrauterine device) OR (depot medroxyprogesterone acetate/DMPA) OR (risk factor)) AND Language = (English) AND Species = (Humans) AND Gender = (Female).

### Study Selection & Data Collection

The studies were reviewed and information extracted by two authors independently (LAV & SW); disagreements were resolved by discussion with CSB and consensus reached. An independent researcher (CEB) identified potential studies from the reference lists of all selected papers for further review. These were then reviewed by LAV & CSB.

We developed a data extraction sheet (based on the Cochrane Consumers and Communication Review Group’s data extraction template) and pilot tested and refined it accordingly. LAV extracted data from included studies and CSB checked extracted data. Consensus for discrepancies was reached by discussion between LAV and CSB, and consultation with JSH and ML as required. Clarification was sought from authors where there was insufficient data to examine the association between HC-use and BV, however this association had clearly been analysed. This included requests for raw data to enable stratification of BV outcome by HC-use. Eight authors were contacted via email, all responded and four were able to provide additional data.

### Data Items

Information was extracted from each included study on: 1) participant characteristics (age, diagnosis method, geographical location, sample size); 2) recruitment setting (sexual/reproductive health service (SRHS), general community healthcare service (GCHS), population-based study (POP), sex worker service (SWS); 3) number of women positive for BV and BV outcome measure (prevalent/incident/recurrent BV); 4) the proportion of women using HC and type of HC method(s) used; and 5) study endpoint definition and length of follow-up for longitudinal studies. HC-use was classified as i) combined (combined oral contraceptive pill [COCP], NuvaRing®) ii) POC (depot medroxyprogesterone acetate [DMPA], implants, injections and norethisterone oenanthate [Net-EN], Mirena®) and iii) any unspecified-HC (including all HC-types listed above but data unavailable to sub-classify as combined or POC).

Studies that utilised duplicated datasets were given the following priority in selection for the meta-analysis to avoid correlation of associations: 1) studies where data was presented stratified by HC-type were given preference over studies with only non-stratified data; 2) studies with adjusted analyses were given preference over those with unadjusted analyses; 3) studies utilising the whole dataset were given priority over sub-studies; and 4) recent publications were used in preference over older publications. Studies that comprised specific populations such as all sex workers, all women who douche, injecting drug users or women diagnosed with concurrent herpes-simplex virus-2 (HSV-2) were included in the analysis.

### Risk of Bias within Individual Studies

We conducted an analysis of the quality of reporting bias using definitions and classifications based on the MOOSE, STROBE and QATSO guidelines [20]–[22] as well as criteria published by Caldeira et al [23]. We assessed the risk of bias within observational studies and RCTs and reported on the following parameters; 1) Have the eligibility criteria and the sources and methods of selection of participants been provided (observational and RCT); 2) For longitudinal studies (cohort and RCT), do they describe the methods of follow up; 3) do the authors report a clearly defined and accepted method of outcome assessment; 4) is there a clearly defined exposure (HC-use) assessment; 5) Is HC-use provided stratified data by HC-type; and 6) have outcome adjustments been performed, particularly for a variable consisting of condom use. Allocation, concealment, blinding and randomisation were not relevant as quality measures in manuscripts using data from RCTs because the data contributing to this meta-analysis was derived from the overall trial (data from the arms was combined).

#### Statistical analyses

We used STATA (Version 12; StataCorp, College Station, TX, USA) for all analyses. For studies that did not report them, 95% confidence intervals (CIs) were calculated using exact methods. Meta-regression using random-effects and p-values for linear trend were used to determine pooled BV prevalence estimates and 95% CIs for eligible studies with sufficient data. To explore variability in study outcome measures (heterogeneity) we hypothesized that the BV prevalence effect size may differ according to the geographical location of the study, the diagnostic method used, and/or the recruitment setting i.e. STI service versus population based studies. Therefore, BV prevalence was stratified by diagnostic method, country group (Europe/UK, Australia/Asia/India, Africa/Middle East, North America, South America) and recruitment setting (SRHS/GHRS/POP/SWS) because of the expected heterogeneity in populations sampled. Given the scope of this review, a summary of this analysis is included in this paper, and further information is available in supplementary material.

The I^2^ test was used to estimate the proportion of total variability in point estimates attributed to heterogeneity other than that due to chance (values of <25%, 25–75%, and >75% representing low, medium, and high heterogeneity, respectively). If the I^2^ statistic was <25%, a meta-analysis based on a fixed-effect model was conducted, otherwise the random-effects (re) model was used. If the I^2^ was >75%, the studies were not combined.

### Primary Summary Measures

The primary outcome measure was the association between any HC-use and prevalent, incident or recurrent BV. For studies where raw data was provided, odds ratios (ORs) and risk ratios (RRs) were calculated using STATA, otherwise estimates and adjusted estimates were used as reported. The meta-analyses were performed by computing pooled unadjusted/adjusted ORs or unadjusted/adjusted relative RRs using either fixed or random-effects models. Quantitative analyses were initially performed for the effect of any HC-use and prevalent, incident and recurrent BV separately, and the composite BV outcome measure (prevalent/incident/recurrent BV), as described below. The secondary outcome measure was the association between specific types of HC-use and each BV outcome measure.

Possible reasons for heterogeneity were explored using pre-specified variables to minimise spurious findings. Variables evaluated included i) BV outcome measure by study-design (prevalent, incident and recurrent BV), and ii) HC-type used in prevalent and incident studies (categorized as combined, POC and any unspecified-HC). We calculated pooled summary statistics of estimates using either fixed or random-effects models depending on the I^2^ statistic, as described above. When statistical heterogeneity was noted, it was evaluated by fitting random-effects meta-regression models to the log-transformed individual study point estimates.

We then determined the association between HC-use and a composite BV outcome measure (prevalent/incident/recurrent BV) in addition to our primary and secondary outcome measures described above. To do this, we first converted ORs to RRs in studies where raw data was available using STATA. For four studies in which raw data was not available [24]–[27], we calculated an approximate RR by extrapolating data provided in the manuscripts to estimate an assumed control group risk and the number of women who were exposed or not exposed to HC. Using the calculated RRs, we then calculated a pooled summary estimate using random-effects models depending on the I^2^ statistic and evaluated statistical heterogeneity by fitting meta-regression models, as described above.

#### Sensitivity analyses

Sensitivity analysis was also performed whereby pooled estimates were calculated omitting one study at a time. We then determined the effect of sub-groups of studies on the pooled estimates by re-estimating the overall effect size after omitting the following sub-groups from the meta-analyses: 1) all studies which recruited women to an RCT (regardless of whether or not the associations were derived from baseline pre-screening data or not); 2) studies in which all women were sex workers; 3) studies in which all women were either sex workers, douched, were injecting drug users (IDUs) or had herpes simplex virus 2 (HSV2); 4) studies in which the HC-use comparison group contained women not using any contraception and/or had undergone tubal ligation; and 5) studies which defined their outcome measure as abnormal flora (NS = 4–10) instead of BV (NS = 7–10).

### Risk of Bias across Studies

We assessed the potential presence of publication bias in studies reporting prevalent and incident BV in separate funnel plots. Asymmetry was statistically evaluated using the Egger’s correlation tests by regressing the log of the estimate (unadjusted/adjusted OR *or* RR) by the log of the standard error (SE) of the estimate. For studies where raw data was not reported, the SE was estimated based on the width of the reported confidence interval using the formula (ln[upper limit of CI]-ln[OR *or* RR])/1.96. The few studies reporting the association between HC-use and recurrent BV were not included in the bias analyses.

## Results

### Study Selection

**Figure 1:**
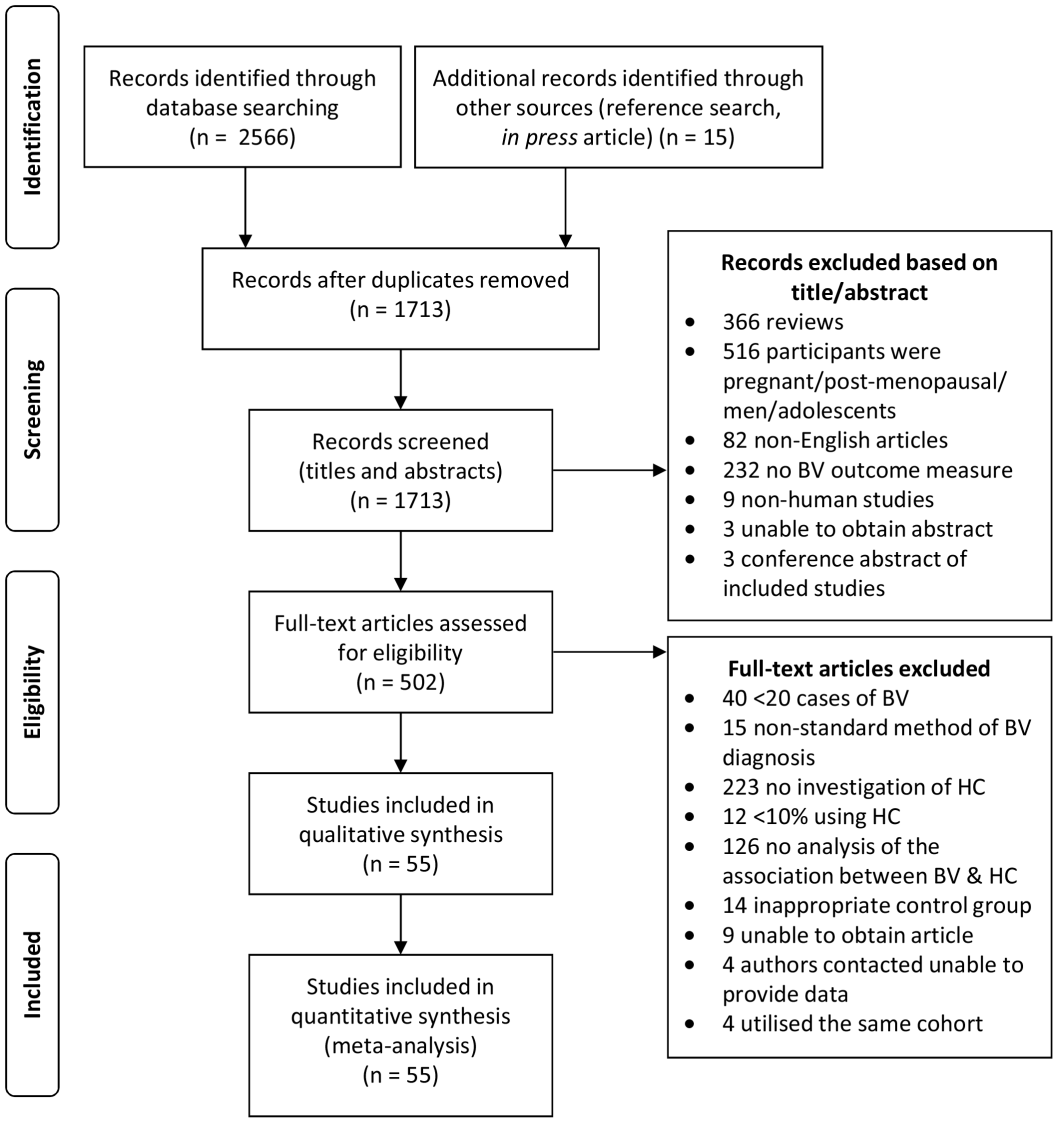
Flowchart demonstrating selection of studies for the systematic review and meta-analysis of the association between hormonal contraceptive (HC) use and bacterial vaginosis (BV).

The review process is outlined in Figure 1 and included papers summarised in Table 1. There were 2566 studies identified from initial searches of Medline (n = 878), Web of Science (n = 997) and Embase (n = 697), 14 additional articles were identified by searching reference lists, and one of our own articles that was *in press* at the time of database searching was included. After removing duplicate articles, 1713 remained of which all titles and abstracts were assessed for potential full text articles to be read; 1211 studies were excluded based on their title and abstract (Figure 1). The full text of 502 articles was reviewed for eligibility criteria, of which there were 59 unique studies that were included in the meta-analysis. Studies were excluded on the basis of: <20 cases of BV (n = 40); use of non-standard methods of BV diagnosis (n = 15), no investigation of HC-use (n = 223), <10% of women using HC (n = 12), there was no analysis of the association between BV and HC-use presented in the manuscript (n = 126), inappropriate/no control group (n = 14), unable to obtain full-text articles (n = 9), and contacted authors were unable to provide data (n = 4).

**Table 1:** Characteristics of prospective studies included in the systematic review.

Reference	Study country	Study design	BV outcome measure	HC-type used	BV diagnostic method	Sample size	% BV positive	% using HC	HC-use comparison group	Unadjusted OR/RR (95% CI)	Reported^a^ Adjusted OR/RR (95% CI)
Amsel 1983 [75]	USA	CS	prevalence	COC	Amsel	311	22.2	26.0	non-combined/NC	0.91 (0.46–1.74)	
Lefevre 1988 [77]	France	CS	prevalence	COC	Amsel	370	26.0	38.9	non-HC/NC	0.72 (0.43–1.21)	
Barbone 1990 [45]	USA	RCT/LC	incidence	COC	modified Amsel	818	38.8	75.4	non-combined/TL		0.84 (0.63–1.10)^b^
Moi 1990 [78]	Sweden	CS	prevalence	COC	Amsel	3762	26.0	53.0	non-combined/NC	0.73 (0.63–0.85)	
Hillier 1991 [79]	USA	CS	prevalence	COC	Amsel	630	32.4	35.1	non-combined/NC	0.62 (0.42–0.90)	
Hart 1993 [58]	Australia	CS	prevalence	COC	modified Amsel	4341	16.9	41.3	non-combined/NC	0.85 (0.72–1.00)	
Cohen 1995 [38]	Thailand	CS	prevalence	COC	Amsel	144	34.0	61.8	non-combined/NC	0.44 (0.21–0.96)	0.50 (0.20–1.20)
				POC	Amsel	144	34.0	14.6	non-POC/NC	1.15 (0.34–3.93)	
Shoubnikova 1997 [12]	Sweden	CS	prevalence	COC	Amsel	956	13.7	69.0	non-combined/NC	0.51 (0.35–0.77)	0.40 (0.20–0.80)
Guerreiro 1998 [80]	Portugal	CS	prevalence	u-HC	modified Amsel	781	7.55	52.0	non-HC	0.20 (0.13–0.49)	
Zenilman 1999 [46]	USA	CS	prevalence	COC	Amsel	272	30.5	20.6	non-combined/NC	0.63 (0.29–1.29)	
Calzolari 2000 [14]	Italy	CS	prevalence	COC	Amsel	1314	16.3	12.6	non-combined/NC	0.47 (0.25–0.82)	0.43 (0.22–0.76)
Baeten 2001 [18]	Kenya	LC	incidence	COC	Nugent	948	39.0	15.5^c^	NC/TL		0.80 (0.70–1.00)
				POC	Nugent	948	39.0	17.8^c^	NC/TL		0.70 (0.50–1.00)
Fonck 2001 [40]	Kenya	RCT/CS	prevalence	COC	Nugent	431	49.0	27.8	non-combined/NC	1.36 (0.88–2.11)	
Holzman 2001 [59]	USA	CS	prevalence	u–HC	Nugent	496	30.0	37.3	non-HC/NC	0.47 (0.28–0.81)	0.50 (0.20–0.80)
Joesoef 2001 [57]	Indonesia		prevalence	u-HC	Nugent	357	32.5	47.3	non-HC/NC	0.60 (0.37–0.96)	
Dan 2003 [81]	Israel	CS	prevalence	COC	Nugent	176	24.0	14.8	non-combined/NC	0.62 (0.24–1.47)	
Ness 2003 [29]	USA	CS	prevalence	u-HC	Nugent	1135	64.0	44.1	non-HC/NC	0.52 (0.40–0.67)	0.60 (0.40–0.80)
Yen 2003 [13]	USA	CS	prevalence	u-HC	Nugent	1938	27.0	36.8	non-HC/NC	0.75 (0.60–0.94)	0.78 (0.62–0.98)
Grio 2004 [82]	Italy	CS	prevalence	COC	Amsel	5230	7.95	17.7	non-combined/NC	0.75 (0.56–1.01)	
Schwebke 2004 [44]	USA	RCT/CS	prevalence	u-HC	Nugent	250	44.8	36.4	non-HC/NC	0.71 (0.41–1.24)	
Smart 2004 [83]	Australia	CS	prevalence	u-HC	Spiegel	1780	5.7	30.6	non–HC/NC	0.67 (0.54–0.83)	0.60 (0.51–0.81)
Watcharotone 2004 [24]	Thailand	CS	prevalence	u-HC	Amsel	800	14.6	41.9	NC/TL	0.97 (0.50–1.90)^b^	
Ashraf-Ganjoei 2005 [25]	Iran	CS	prevalence	COC	Amsel	130	37.7	N/A^d^	non-HC/NC	0.37 (0.14–0.99)^b^	
Bradshaw 2005 [15]	Australia	CS	prevalence	COC	Amsel/Nugent	342	46.0	46.8	non-HC/NC	0.62 (0.39–0.97)	0.60 (0.40–1.00)
Harville 2005 [48]	USA	CS	prevalence	u–HC	Nugent	411	26.3	42.1	non–HC/NC	0.61(0.37–0.98)	
Plitt 2005 [43]	USA	RCT/CS	prevalence	COC	Nugent	115	60.0	20.9	non-combined/NC	1.05 (0.38–3.04)	
Schwebke 2005 [47]	USA	LC	incidence	u-HC	Nugent	96	69.8^a^	41.7^e^	non-HC/NC	0.49 (0.17–1.40)^b^	
Bradshaw 2006 [10]	Australia	LC	recurrence	u-HC	Nugent	122	55.7^a^	38.7 e	non-HC/NC	0.40 (0.20–0.80)^b^	0.50 (0.30–1.00)
Amaral 2007 [35]	Brazil	CS	prevalence	u-HC	Nugent	155	75.5	44.5	non-HC/NC	0.56 (0.25–1.26)	
Cauci 2007 [84]	Italy	CS	prevalence	COC	Nugent	500	29.0	27.2	non-combined/NC	0.49 (0.29–0.80)	
Evans 2007 [85]	UK	CS	prevalence	u-HC	Ison-Hay	169	14.4	50.3	non-HC/NC	0.77 (0.29–2.00)	
Hassan 2007 [36]	Africa	RCT/CS	prevalence	POC	Nugent	237	33.8	24.5	non-POC/NC	0.76 (0.38–1.51)	
Kleinschmidt 2007 [51]	South Africa	CS	prevalence	POC	Nugent	551	34.7	54.4	non-HC/NC	0.96 (0.67–1.38)	
Koumans 2007 [30]	USA	CS	prevalence	COC	Nugent	3739	29.2	10.4	non-combined/NC		0.65 (0.40–1.00)
Riggs 2007 [16]	USA	CS	prevalence	COC	Nugent	8542^f^	40.0^f^	29.4 f	non-combined/NC	0.64 (0.55–0.74)^b^	0.76 (0.63–0.9)
				POC	Nugent	8542^f^	40.0^f^	25.8 f	non-POC/NC	0.76 (0.66–0.88)^b^	0.64 (0.53–0.76)
		LC	incidence	COC	Nugent	2636		31.2 f	non-combined/NC	0.92 (0.73–1.17)^b^	1.13 (0.88–1.43)
				POC	Nugent	2636		24.4 f	non-POC/NC	1.04 (0.78–1.39)^b^	1.07 (0.79–1.45)
		LC	recurrence	COC	Nugent	2892	84.2	21.5 f	non-combined/NC	0.68 (0.53–0.87)^b^	0.76 (0.56–1.03)
				POC	Nugent	2892	84.2	19.0 f	non-POC/NC	0.61 (0.48–0.79)^b^	0.6 (0.44–0.81)
Cherpes 2008 [52]	USA	LC	incidence	COC	Nugent	773	36/100^g^	62.9^g^	NC	0.80 (0.60–1.10)^b^	
				POC	Nugent	773	36/100^g^	280.7^g^	NC	1.20 (0.80–1.90)^b^	
Mares 2008 [86]	Brazil	CS	prevalence	COC	Nugent	40	57.5	12.5	non–combined/NC	0.48 (0.04–4.79)	
				POC	Nugent	40	57.5	22.5	non–POC/NC	0.90 (0.16–5.51)	
McClelland 2008 [37]	Kenya	RCT/LC	incidence	POC	Nugent	151	37.1	28.5	NC/TL	0.59 (0.48–0.73)^b^	0.60 (0.48–0.74)
Peipert 2008 [60]	USA	RCT/CS	prevalence	u-HC	Amsel/Nugent	523	31.0	69.0	non-HC/NC	0.75 (0.48–1.16)	
Rugpao 2008 [53]	Thailand	LC	incidence	COC	Amsel	1522	10^b^	35.9	non-combined/NC	1.00 (0.75–1.33)^b^	
			incidence	POC	Amsel	1522	10^b^	42.1	non-POC/NC	0.77 (0.57–1.02)^b^	
Baisley 2009 [42]	Tanzania	CS	prevalence	u-HC	Nugent	1305	62.9	46.2	non-HC/NC	0.72 (0.56–0.93)	0.80 (0.62–1.04)
Fethers 2009 [55]	Australia	CS	prevalence	COC	Nugent	528	4.7	40.2	non-combined/NC	0.82 (0.32–1.99)	
Pettifor 2009 [49]	South Africa	LC	incidence	POC	Nugent	567	35.6	19.9	non-POC/NC	0.75 (0.55–1.02)^b^	0.77 (0.56–1.06)
Rifkin 2009 [27]	USA	CS	prevalence	COC	Amsel	330	40.3	58.2	NC/TL	1.01 (0.67–1.52)^b^	0.66 (0.39–1.10)
				POC	Amsel	330	40.3	17.0	NC/TL	0.42 (0.24–0.74)^b^	0.42 (0.20–0.88)
Tibaldi 2009 [26]	Italy	CS	prevalence	COC	Amsel	24122	8.9	16.5	non-combined/NC	0.86 (0.72–1.04)^b^	0.69 (0.56–0.85)
Yotebieng 2009 [39]	Thailand	RCT/CS	prevalence	u-HC	Amsel	901	57.0	24.9	non-HC/NC	0.46 (0.34–0.64)	
Brotman 2011 [56]	USA	CS	prevalence	u-HC	Amsel	93	67.0	12.9	non-HC/NC	1.00 (0.24–4.95)	
Bukuski 2011 [50]	Kenya	RCT/LC	recurrence	u-HC	Nugent	164	42.7	33.5	non-HC/NC	1.11 (0.77–1.60)	
Kampan 2011 [87]	Malaysia	CS	prevalence	u-HC	Amsel	131	19.1	49.6	non-HC/NC	0.86 (0.32–2.23)	
Gallo 2012 [54]	USA	LC	incidence	COC	Amsel	645	39.5	29%^h^	non-combined/NC	0.67 (0.45–0.91)^b^	0.59 (0.42–0.91)
Mascarenhas 2012 [88]	Brazil	CS	prevalence	u-HC	Nugent	100	20.0	41.0	non-HC/NC	0.95 (0.30–2.86)	
Perla 2012 [41]	Peru	CS	prevalence	COC	Nugent	212	44.8	10.8	non-combined/NC	0.62 (0.23–1.66)	
Bradshaw 2013a [17]	Australia	RCT/LC	recurrence	COC	Nugent	404	28.0	27.2	non-combined/NC	0.63 (0.41–0.96)	0.52 (0.34–0.81)
Bradshaw 2013b [7]	Australia	CS	prevalence	COC	Nugent	1093	11.8	53.5	non-combined/NC	0.60 (0.40–0.80)^j^	0.60 (0.40–0.90)
		LC	incidence	COC	Nugent	864	11.8	53.5^i^	non-combined/NC	0.70 (0.50–1.00)^b^	0.70 (0.50–1.10)

a all adjusted OR/RRs are as reported by authors,

b OR/RRs calculated by authors, raw data not available,

c baseline prevalence used,

d raw data on the % using OC not available, on the basis of the odds ratios reported, the proportion of women using contraceptives were calculated to well exceed 10%,

e per number of visits,

f number or percentage of assessments rather than number of participants reported, ^g^woman-years, calculated by authors,

h raw data was not available, but parent study from which this cohort was derived reported 29% using HC [89],

i baseline prevalence of women using combined methods of contraception was 53.5% of which most went into the longitudinal analysis, but raw data was not reported,

j unadjusted ORs clustered by clinic so included as reported. Key: HC = hormonal contraception, COC = combined oestrogen- and progesterone-containing methods of HC, POC = progesterone only containing methods of HC, u-HC = unspecified HC, CS = cross-sectional study, LC = longitudinal cohort, RCT = randomised controlled trial (either LC used or CS data used), OR = odds ratio, RR = risk ratio, NC = no contraception used, TL = tubal ligation.

Three cohorts of women were utilised for more than one manuscript. To avoid the issue of increased weighting of these cohorts in the meta-analysis we therefore applied our hierarchical selection criteria to select only one publication for each of the three cohorts. The two studies by Ness et al [28], [29] utilised data from women enrolled in the GYN Infections Follow-through study. Based on our systematic approach to selection the most recent study was included in our review and meta-analysis. Both Koumans [30] and Hensel [31] reported associations with BV using data from women enrolled in the NHANES cohort. Hensel only reported on a sub-set of the cohort so was excluded and Koumans was included. Finally, Nansel et al 2006 [32], Riggs et al 2007 [16] and two studies by Klebanoff [33], [34] all used data from women enrolled in Longitudinal Study of Vaginal Flora cohort to determine the association between HC-use and prevalent and incident BV. Only Riggs stratified HC-use by combined HC-use and POC-use so only this study was included and the others were excluded from our meta-analysis. These exclusions meant only 55 of the 59 unique publications were included in the systematic review and meta-analysis.

### Study Characteristics

All 55 studies included in the analysis were peer-reviewed English language original articles. These studies used the following BV diagnostic methods: standard Amsel methods (n = 18), modified Amsel method (n = 3), Nugent method (n = 30), both Amsel and Nugent (n = 1), Spiegel method (n = 1) and Ison-Hay criteria (n = 1) (Table 1).

Studies using the Nugent method differed in whether they compared women with a NS = 7–10 (established Nugent definition of BV) to all other participants [NS = 0–6 (n = 29)] or they excluded women with intermediate flora (NS = 4–6) and compared NS = 7–10 to NS = 0–3 (n = 4). Two studies reported comparison of women with abnormal flora (NS = 4–10) to normal flora (NS = 0–3) [29], [35], and one study defined BV as women with a NS = 7–10 or NS = 4–6 with ≥3 Amsel criteria [15]. Data was included as reported in the publications for these studies and did not have significant implications for the association as determined by sensitivity analyses (see below and Table S1, Table S2 and Table S3).

#### Outcome measure

Studies were able to contribute >1 set of data to the analysis if they reported on both combined and POC HC-types separately or >1 BV outcome (prevalent and incident BV), Table 1. For example, 43 studies contributed 47 associations between HC-use and prevalent BV, 10 contributed 14 associations between HC-use and incident BV, and 4 studies contributed 5 associations between HC-use and recurrent BV. For HC-use, one study reported each association between combined HC-use and POC-use and prevalent, incident and recurrent BV separately [16], and five studies reported each association between BV and combined and POC methods separately. One reported each association between prevalent BV and unspecified HC-use and combined HC-use separately, but only the association of BV and combined HC-use was included [7]. In two of the included studies in which the association between both POC and combined HC-use and prevalent BV [36] and incident BV [37] were investigated separately from the same cohort, only POC-use was included in the meta-analysis, as less than 10% of the population were using a combined HC method.

The majority of eligible studies were cross-sectional and North American. A minority of studies contained specific population sub-groups: in eight all participants were sex workers [18], [35]–[41], in one all had HSV-2 [42], in one all were IDUs [43], and in another all douched [44]. In several studies, participants were predominantly African-American or sub-Saharan African, including eight North American studies [16], [27], [29], [45]–[48] and nine African studies [18], [36], [37], [39], [40], [42], [49]–[51]. Although studies where all women were menopausal were excluded, eight included women of an age where some were or may have been peri- or post- menopausal.

Incidence was defined differently in the ten incident BV studies. Time to first BV diagnosis was defined as incident BV in 5 studies, at which point women were censored [7], [45], [47], [52], [53]. Three of these studies reported 4-monthly follow-up for up to 12 months [7], [47], [52]; Barbone reported monthly follow-up for up to 6 months [45], Rugpao reported 3-monthly visits for 15–24 months [53]. Three studies defined incident BV as a negative visit, followed by a consecutive positive visit [16], [49], [54], but did not censor at first incident diagnosis, and women could contribute data multiple times during 6–12 months of follow-up. In two studies, multiple and successive BV diagnoses from an individual were deemed to be new discrete incident cases of BV, however some of these cases may have reflected persistent infection [18], [37].

The four studies reporting recurrent BV also used differing study endpoints. Women reached study endpoint if they had a repeat BV diagnosis or completed follow-up without BV in the Australian [10], [17] and African [50] studies (6 and 2-months of follow-up, respectively). In the North American study [16], recurrent/persistent BV was defined as having a BV positive visit, followed by a consecutive BV positive visit, so women experiencing more than two consecutive episodes of BV were deemed to have more than one episode of recurrence.

#### BV prevalence

The median prevalence of BV was 32.4% and ranged from 4.7% in asymptomatic 17–21 year old Australian university students [55] to 66.7% of American women enrolled in a sub-study [56] (Figure S1). The I^2^ was >75% so studies could not be combined for overall pooled estimates. We stratified BV prevalence by: i) geographical location, ii) BV diagnostic method, and iii) recruitment setting. The median BV prevalence was higher from North America (33.2% range; 16.1–66.7%), South America (47.9%; 20.0–57.5%) and Africa (37.7%; 33.8–62.9%) compared with Europe and the UK (14.4%; 7.6–29.0%), Asia and Australia (16.9%; 4.7–46.0) (Table 2, Figure S1), and slightly higher in studies that employed the Nugent (35.6%; 4.7–62.9%) compared to Amsel (22.2%; 7.6–66.7%) method (Table 2, Figure S2). Stratifying BV prevalence by recruitment setting also demonstrated variation in reported BV prevalence (Table 2, Figure S3). This suggests that variation in BV prevalence is likely to be due to differences in geographic location, diagnostic methods used, and recruitment settings.

**Table 2:** Assessment of bias: measures of the studies included in the analysis.

Measure	Variables	N-studies
BV outcome measure	prevalence	43^a^
(55 studies, 57 outcomes)	incidence	10^a^
	recurrence	4^a^
Hormonal contraceptive type	Combined HC-use	33^a^
(55 studies, 66 associations)	POC HC-use	13^a^
	Unspecified HC-use	20^a^
BV diagnostic method	Nugent	30^a^
(n = 55 studies)	Amsel (modified Amsel n = 3)	21^a^
	Amsel & Nugent	1^a^
	Other: Spiegel (n = 1), Ison-Hay (n = 1)	2^a^
Setting/recruitment venue	Sexual or reproductive health service (SRHS)	37^b^
(n = 55 studies)	General community healthcare service (GCHS)	7^c^
	Sex worker service (SWS)	8^d^
	Population based (POP)	2^e^
HC-use comparison group	no contraceptive use	8^a^
(n = 66 associations)	all non-hormonal contraceptives or none	23^a^
	all non-combined contraceptives or none	27^a^
	all non-progesterone contraceptives or none	8^a^

a references for first three summary measures provided in Table 1; references for the last two summary measures footnoted here:

b [10], [12], [14], [15], [17], [24]–[27], [29], [45]–[54], [57], [58], [60], [75], [77]–[88],

c [7], [13], [16], [42]–[44], [59],

d [18], [35]–[41].

e [30], [55].

Key: HC = hormonal contraception, combined = combined oestrogen- and progesterone-containing methods of HC, POC = progesterone only containing methods of HC.

### Risk of Bias within Studies

Most observational studies, including all RCTs, were considered to have adequately reported inclusion and exclusion criteria and to have provided justification for selection of participants. Three studies included all women without specifying specific exclusion criteria [25], [26], [57], [58]. For all longitudinal studies, the methods of follow-up were described. Due to our quality criteria for inclusion, all studies clearly defined the method of outcome assessment (Amsel, Nugent, Spiegel or Ison-Hay), with only two studies defining their outcome measure as abnormal flora (NS 4–10) instead of BV (7–10) [29], [35], as previously described. The measurement of exposure to HC in each study was clearly defined, with most studies reporting on current use of HC at the time of assessment. However, five studies included HC-use as any reported use in a time period that ranged from 3–6 months [17], [43], [48], [53], [59]. Twenty-two studies did not stratify HC-use by combined HC-use or POC-use, and so were included as unspecified HC-use. This unspecified HC group may have been a source of bias within studies as it could have predominantly contained only one specific subtype of HC users. The comparison groups varied between studies, but participants not using HC were the comparison group in half the studies (Table 1). In 8 studies, the comparison group included women who were not using any contraception and/or who had undergone tubal ligation. Sensitivity analyses excluding studies where the control group was exclusively women not using any contraception were conducted to examine whether this influenced the overall estimates (Tables S1, S2 and S3). No evidence of bias from inclusion of these studies was found. Twenty-nine studies reported results adjusted for at least one confounder. Of these, 23 adjusted for condom use, 13 for age, one for all other variables assessed in the study and one study [14] performed a stepwise logistic regression to identify possible confounders, but did not state which variables were included in the final multiple regression.

Allocation, concealment, blinding and randomisation were not relevant as quality measures in manuscripts using data from RCTs because the data contributing to this meta-analysis was derived from the whole trial (data from the arms was combined), for all but one study which only used women in the placebo arm (*vs* vaginal presumptive treatment arm) [37]. Importantly, all of the included RCTs are of different interventions and heterogeneous populations. One RCT used pre-trial/screening data so individuals that were analysed in the dataset were not highly selected and subject to rigorous exclusion/inclusion criteria [40]. All others used “on-trial” baseline or longitudinal data, however the trials conducted were of a diverse range of interventions and included an antibiotic/probiotic trial [17], spermicide trial [45], presumptive prophylaxis with 1g azithromycin [40] or metronidazole and fluconazole [36], [37], behavioural intervention [43], [60], counselling intervention [39], male partners used an ethanol gel [50], or suppressive acyclovir treatment [42]. The studies that selected specific groups of participants had very diverse selection criteria e.g. all douche [44], all have HSV2 [42], all are IDUs [43]. The highest amount of publication bias was found for potential reporting bias. In 6/11 RCTs results were presented for BV as a pre-specified primary outcome but in 5/11 the primary outcome was a decrease in STIs or risk factors for HIV, and BV analyses were secondary or additional reported outcomes. To address the issue that individuals participating in RCTs may not be entirely representative of the general population and provide a source of within study bias RCTs were excluded as a sub-group in the sensitivity analyses (Tables S1, S2 and S3) and were shown to have no influence on the overall estimates.

### Results of Individual Studies

**Figure 2:**
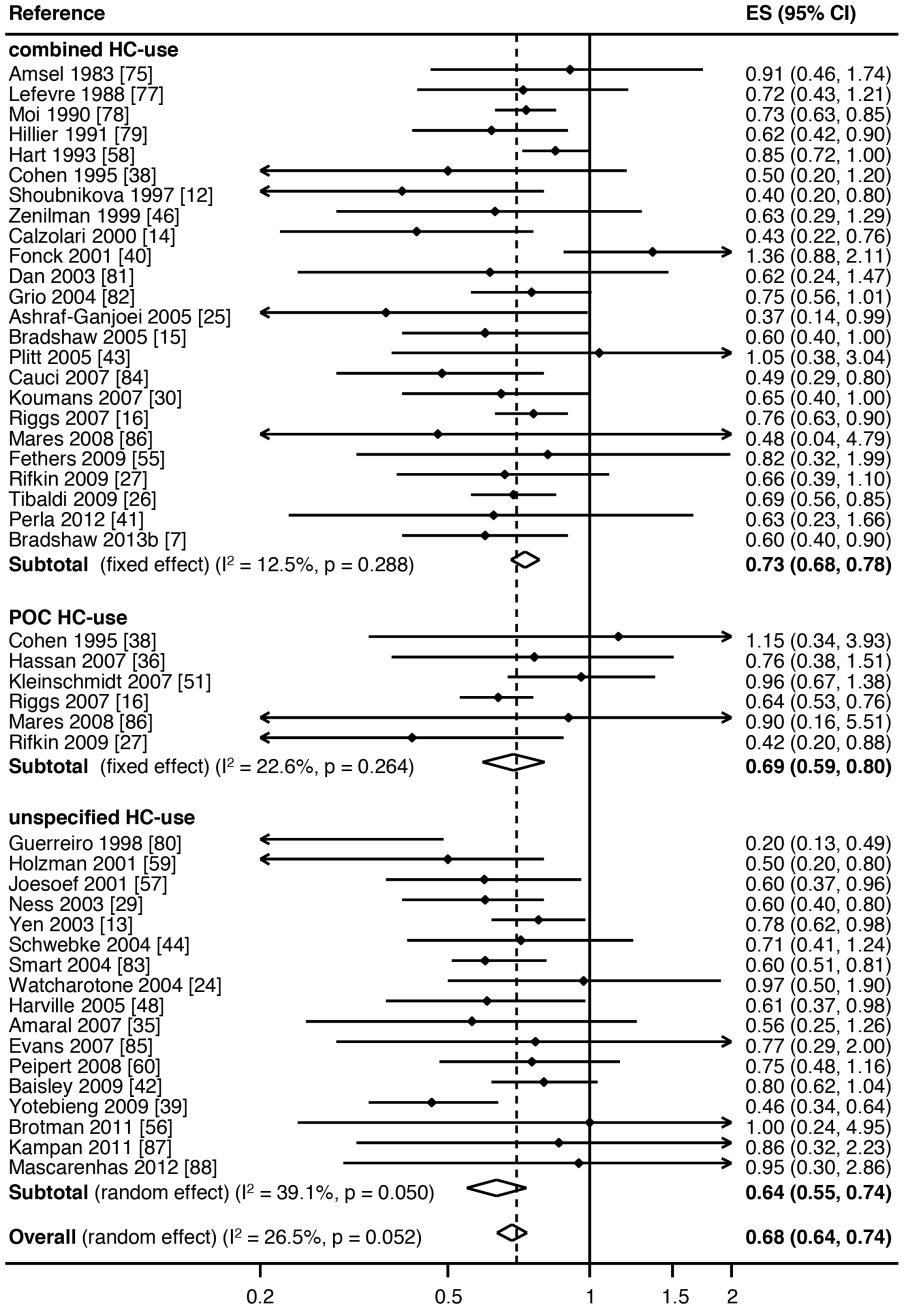
Meta-analysis of the association between hormonal contraceptive (HC) type and prevalent BV. Key: ES = effects size, CI = confidence interval, combined HC-use = combined oestrogen- and progesterone-containing methods of HC, POC-use = progesterone only containing methods of HC.

The association between combined HC-use, POC-use or any unspecified HC-use and prevalent BV was reported in 24, 6 and 17 studies, respectively, Table 1. The average proportion of women using any HC in studies was 34.4% (range 10.4%–75.4%). To reduce the risk of confounding, adjusted ORs/RRs were included where provided. Any HC-use was associated with a significantly reduced prevalence of BV in 19 studies, three studies reported a borderline association with an upper 95%CI of 1.00 (Table 1, Figure 2), and there was no significant association between any HC-use and BV in 25 studies. When stratified by HC-type, of the 24 studies reporting combined HC-use, nine reported a significantly reduced prevalence of BV, three a borderline association (upper 95%CI of 1.00) and 12 reported no significant association. Of the six studies investigating POC-use, only two reported a significantly reduced prevalence of BV. Of the 17 studies examining unspecified HC-use, 8 reported a significantly reduced risk of prevalent BV.

**Figure 3:**
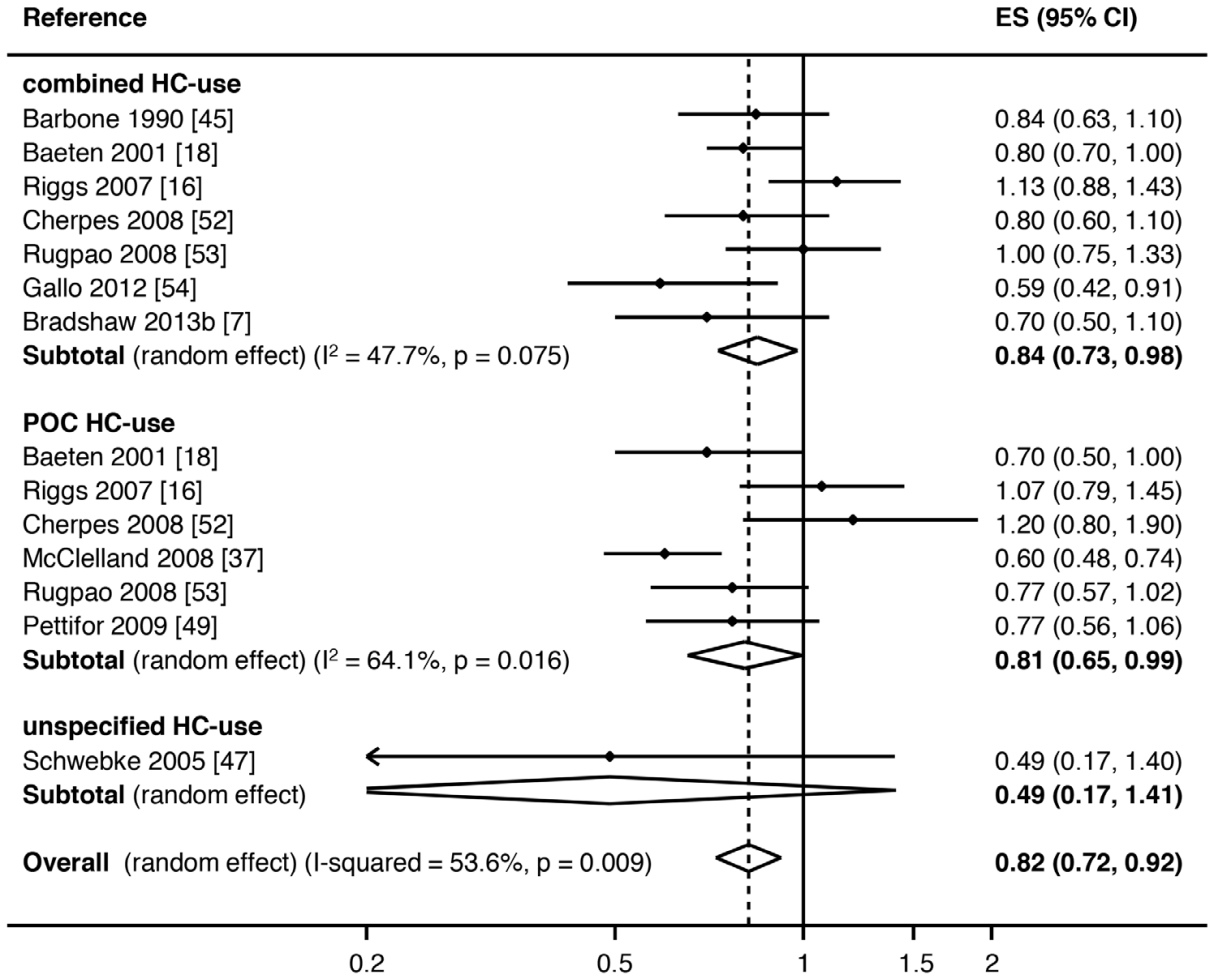
Meta-analysis of the association between hormonal contraceptive (HC) type and incident BV. Key: ES = effects size, CI = confidence interval, combined HC-use = combined oestrogen- and progesterone-containing methods of HC, POC-use = progesterone only containing methods of HC.

**Figure 4:**
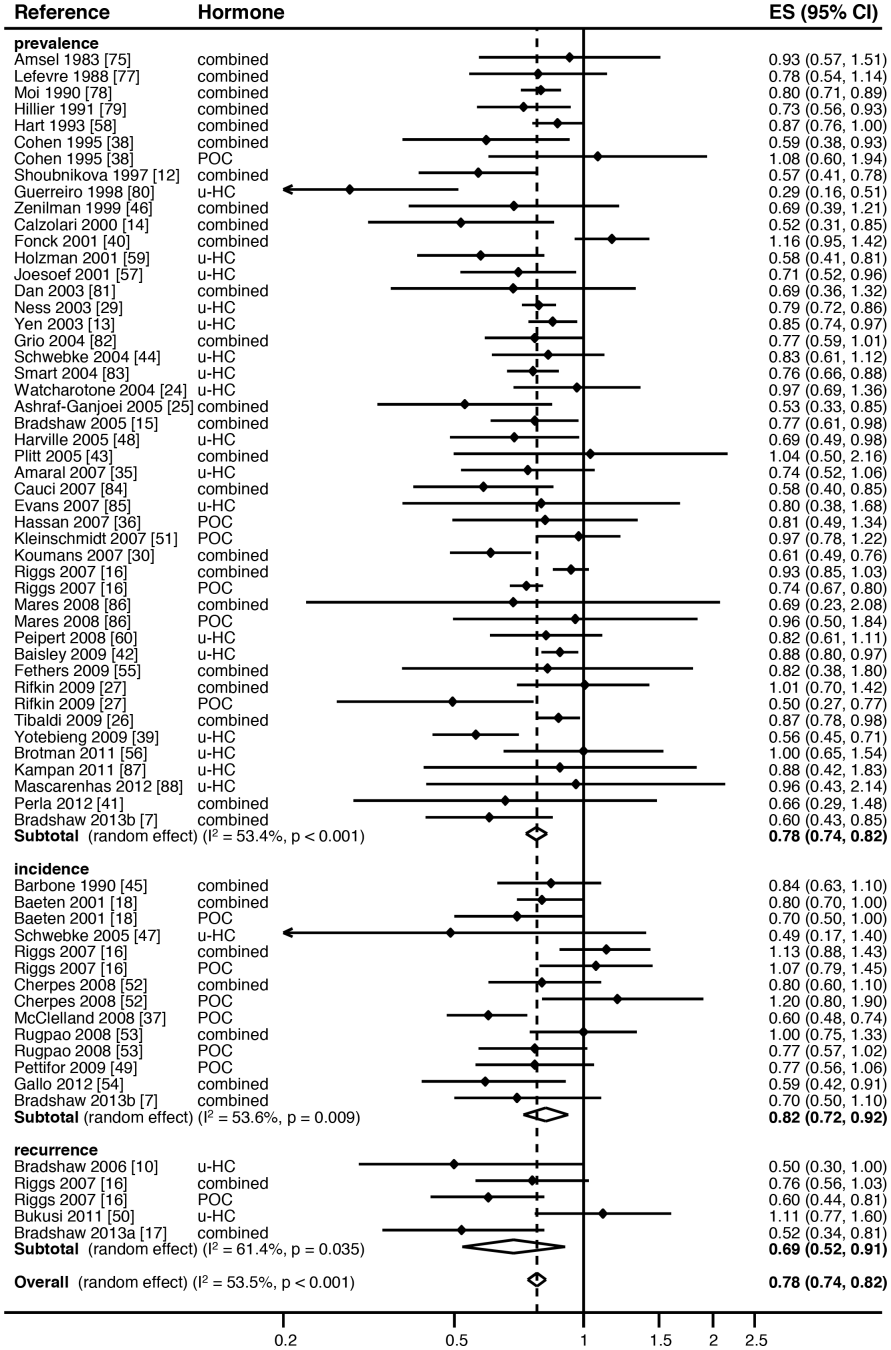
Meta-analysis of the association between specified and unspecified hormonal contraceptive (HC) use and BV outcome, stratified by prevalent, incident or recurrent BV. Key: ES = effects size, CI = confidence interval, combined = combined oestrogen- and progesterone-containing methods of HC, POC = progesterone only containing methods of HC, u-HC = unspecified HC.

Of the 14 incident analyses, 10 showed a reduced risk of incident BV with HC-use (RR<1.00) (Table 1, Figure 3). Only two studies had a significant association with incident BV, one with combined HC-use and one with POC-use. Two studies had a borderline association with incident BV, again one with combined HC-use and one with POC-use. None of the studies using any unspecified HC-type had a significant association with incident BV. In the five recurrent analyses, four showed a decreased risk of recurrent BV in HC-users, which was significant in one study reporting combined HC-use and one study reporting POC-use (Table 1, Figure 4).

### Synthesis of Overall Results

#### Association between BV and hormonal contraceptive use

Due to some studies reporting the association between BV and different types of HC, the 43 prevalence studies contributed 47 datasets or associations, 10 incident studies contributed 14 datasets, and 4 recurrent studies contributed 5 datasets.

Hormonal contraceptive use was associated with a significant reduction in the odds of prevalent BV (pooled effect size by random-effects [reES] = 0.68, 95%CI:0.63–0.73, p<0.001), with 27% of differences between studies due to heterogeneity (I^2^ = 26.5%, p = 0.05), Figure 2. HC-use was also associated with a significant reduction in the relative risk of incident BV (pooled reES = 0.82, 95%CI:0.72–0.92, p = 0.001), with 54% of differences due to heterogeneity (I^2^ = 53.6%, p = 0.03), Figure 3. In the analysis of the effect of HC-use on recurrent BV, HC-use was associated with significantly decreased risk of recurrent BV (pooled reES = 0.69, 95%CI:0.52–0.91, p<0.001), with 62% of differences between studies due to heterogeneity (I^2^ = 61.6%, p = 0.03).

We then stratified type of HC-use (combined/POC/unspecified HC) by either prevalent or incident BV (Figures 2 and 3, respectively). With ≤2 studies contributing to the association between recurrent BV and specific HC-types in the meta-analysis, further sub-group analysis was not feasible for recurrent BV. Combined HC-use was associated with a significantly decreased odds of prevalent BV (pooled ES = 0.72, 95%CI:0.66–0.78, p<0.001), with 11% of observed variance due to heterogeneity between studies (I^2^ = 12.5%, p = 0.29). POC-use was also associated with significantly decreased rate of prevalent BV (pooled ES = 0.69, 95%CI:0.59–0.80, p<0.001), with 23% of observed differences due to heterogeneity (I^2^ = 22.6%, p = 0.26). Unspecified HC-use was also associated with significantly decreased rate of prevalent BV (pooled reES = 0.64, 95%CI:0.55–0.74, p<0.001), with 39% of observed variance explained by heterogeneity (I^2^ = 39.1%, p = 0.05). Meta-regression analysis revealed no heterogeneity between HC-types (overall p = 0.43; combined v POC p = 0.82, combined v u-HC p = 0.25).

When the association between different sub-groups of HC-use and incident BV was examined, both combined HC-use and POC regimens were associated with a decreased risk of incident BV, with pooled reES of 0.85 (95%CI:0.73–0.98, p = 0.02) and 0.81 (95%CI:0.65–0.99, p = 0.04), respectively, Figure 3. Both associations displayed moderate heterogeneity (I^2^ = 47.7%, p = 0.08 and I^2^ = 64.1%, p = 0.02, respectively). There was only one study reporting the association between unspecified HC-use and incident BV (RR = 0.49, 95%CI:0.17–1.40). Meta-regression analysis revealed no heterogeneity between HC-types (overall p = 0.55; combined v POC p = 0.62, combined v u-HC p = 0.31).

In the final meta-analysis we examined the effect of any HC-use on the composite outcome of prevalent/incident/recurrent BV. To generate an overall pooled estimate all ORs had to be converted as described in the methods to RRs. As RRs will always show a smaller effect size than ORs, the association between HC-use and the risk of prevalent BV changed (pooled reES = 0.78, 95% CI:0.74–0.82), Figure 4. For the composite outcome, HC-use was associated with a significant reduction in any BV (pooled reES = 0.78, 95%CI:0.74–0.82, p<0.001), Figure 4. We detected moderate, but significant heterogeneity within this comparison (I^2^ = 53.5%, p<0.001), suggesting that 54% of the observed variance between studies can be explained by heterogeneity. Meta-regression analysis revealed no heterogeneity between studies reporting prevalent, incident and recurrent BV (overall p = 0.43; prevalent v incident BV p = 0.36; prevalent v recurrent BV p = 0.42).

#### Sensitivity analyses

We conducted a range of sensitivity analyses to determine the influence certain studies and specific populations had on the overall estimates. The removal of specific populations, RCTs and individual studies did not qualitatively alter associations (Tables S1, S2 and S3). The confidence intervals around the pooled estimates only crossed 1 after exclusion of some sub-groups when looking at the association between HC-types and incident BV, most likely because of the smaller number of studies contributing to these analyses.

### Risk of Bias across Studies

**Figure 5:**
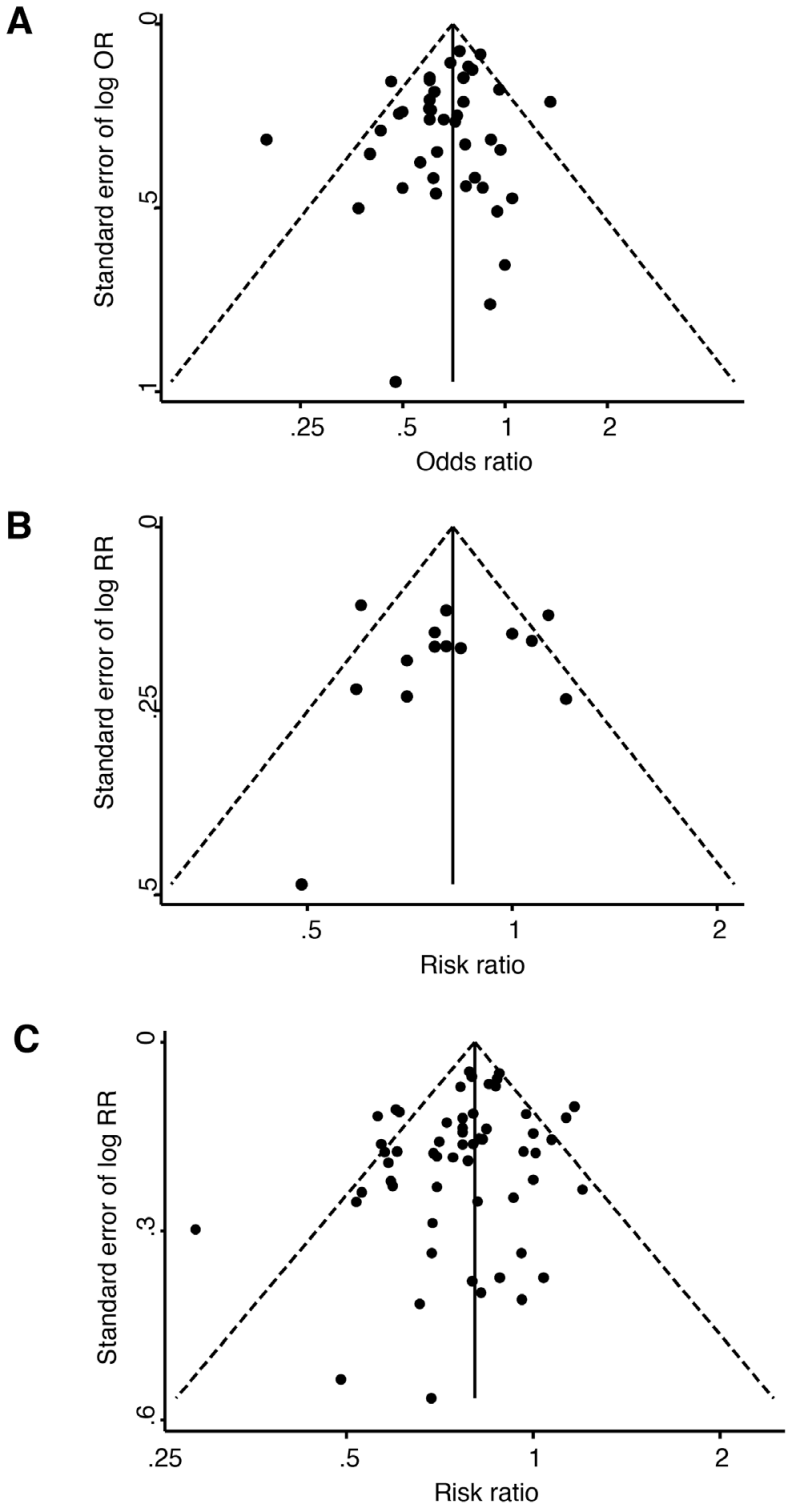
Funnel plots demonstrating the potential presence of publication bias in studies reporting A) prevalent BV, B) incident BV and C) the composite outcome of any BV. Key: OR = odds ratio, RR = risk ratio.

To explore the heterogeneity in the association between HC-use and prevalent or incident BV, funnel plots were drawn using estimates for prevalent and incident BV separately. The funnel plot of the association between any HC-use and prevalent BV showed little asymmetry, with no significant indication for publication bias (Egger’s Bias coefficient = −0.59, 95%CI: −1.31–0.12, p = 0.10), Figure 5A. This observation suggests that publication bias is unlikely. The second funnel plot using RR estimates for incident BV showed more asymmetry, particularly due to the lack of smaller studies, Figure 5B. However, there was also no significant indication of publication bias (Egger’s Bias coefficient = −0.36, 95%CI: −3.38–2.66, p = 0.80). A third funnel plot of the association between any HC-use and composite outcome of any BV showed little asymmetry, again, with no indication of publication bias (Egger’s Bias coefficient = −0.34, 95%CI: −3.31–2.62, p = 0.81), Figure 5C.

## Discussion

This systematic review examined the association between use of hormonal contraceptives and detection of BV and supports the hypothesis that women using HC have a decreased risk of BV, compared to women not using a hormonal method of contraception. This negative association was robust and present regardless of the HC-type reported, and was evident across all three BV outcome measures (prevalent, incident and recurrent BV), with the exception of unspecified HC-use and incident BV, for which there was only one study. Most data were available from prevalence compared to incidence studies, and there were few studies examining recurrence; however HC-use was associated with a statistically significant reduced risk of BV for each of these outcomes when separately examined. Hormonal contraceptive use was associated with a 32% reduction in the odds of prevalent BV (pooled reES = 0.68, 95%CI:0.63–0.73), an 18% reduction in the relative risk of incident BV (reES = 0.82, 95%CI:0.72–0.92), and a 31% reduction in the risk of recurrent BV (reES = 0.69, 95%CI:0.59–0.91). Unexpectedly, when stratified by reported HC-type, combined HC-use and POC methods were associated with a similar magnitude reduction in prevalent BV risk. When all estimates were converted to RRs, the meta-analysis showed that individuals using any HC-type had a significant overall reduction in risk of the composite BV outcome (reES = 0.78, 95%CI:0.74–0.82). This meta-analysis provides compelling evidence that HC-use influences a woman’s risk of BV, with important implications for clinicians and researchers in the field. Importantly, these data encompassed high and low BV prevalence populations in geographically diverse settings, and had a low level of publication bias indicated by funnel plot and Eggers bias tests, and were not influenced by a number of varied sensitivity analyses.

The negative association between HC-use and BV may be somewhat surprising in light of recent data implicating HC, particularly POC, with possible increased risk of HIV transmission [61]. However, over the last 30 years evidence has been emerging from observational studies of a negative association between HC-use and BV. Although the observed association could be due to confounding, it is evident across a large number of studies, many of which attempted to control for the confounding effects of behaviours, including condom use and recent sexual practices. A number of possible biological mechanisms may underlie this observed reduction in BV risk. One plausible hypothesis, that is more relevant to oestrogen-containing contraceptives, is that they may reduce the risk of BV by increasing the glycogen-content of epithelial cells, which is metabolised to lactic acid by epithelial cells and lactobacilli. Lactic acid is thought to be the primary vaginal acidifier and a known potent inhibitor of BV[62]–[64]. Higher lactic acid abundance has been reported in women with a vaginal microbiome dominated by *L.crispatus*, which appear able to produce more lactic acid than other species [65]. Furthermore, lactic acid has also been shown to elicit a favourable cytokine response in the female genital tract [66], which may further assist in reducing the risk of BV.

While the glycogen-lactic acid theory may explain a protective effect from oestrogen, it seems unlikely to be relevant to progesterone-only contraceptives, which often result in an oestrogen-deficient state. Interestingly, however, both progesterone and oestrogen appear to regulate a number of important immune mechanisms in genital tract epithelial and immune cells, with mid-cycle immunological suppression allowing for fertilization and pregnancy. There is direct and indirect cyclical regulation of soluble immune mediators, such as immunoglobulins (in particular IgA and IgG), secretory leukocyte protease inhibitor (SLPI), and defensins, which have antimicrobial actions against bacteria, fungi and viruses. Both sex steroids also influence recruitment of lymphocytes, natural killer cells, macrophages and Langerhans cells and production of cytokines [67], [68]. The actions of these hormones are complex and appear to vary depending on concentration, and to also differ between the vagina and the endometrium [67]. Oestrogen exerts pro-inflammatory effects at low concentrations, and anti-inflammatory effects at high concentrations [69]. The concentrations and cyclical pattern of expression of β-Defensins have been reported to differ during the phases of the menstrual cycle and between combined HC users compared to non-users [70].

A further mechanism by which HC, particularly progesterone-containing HC, may protect against BV is by reducing the frequency of menstruation, and therefore the volume and presence of haemoglobin in the genital tract. A number of studies have reported that BV is detected more commonly at the beginning of the menstrual cycle when oestradiol levels are lowest [15], [59], [71], [72]. Iron is essential for growth for most bacteria, including BVAB. Experiments have shown that *G.vaginalis* is capable of both utilizing iron-containing compounds from sources including haemoglobin, and producing siderophores to acquire iron from the environment [73]. Furthermore, quantities of *L.jensenii* and *L.crispatus* have been shown to decrease and *G.vaginalis* concentrations to increase with the onset of menses [74]. It is possible that through reduction in menstrual loss, HC-use influences susceptibility to colonization with BVAB, and that this effect may be particularly relevant to progesterone-only methods that commonly produce amenorrhoea.

Clearly, further research is needed to understand the complex multifaceted effects of both oestrogen and progesterone on the vaginal environment. However, one could reasonably postulate that increased and sustained circulating levels of sex hormones could potentially act in a number of favourable ways to promote and support a healthy vaginal state and reduce the risk of BV. This may include facilitating growth of protective *Lactobacillus* species, and supporting sustained high levels of lactic acid and favourable alterations to immune mechanisms in the female genital tract, that promotes vigorous host responses and clearance of BVAB. While more research is needed to disentangle the biological mechanisms that may underlie this association, clearly only a randomised controlled trial (RCT) will determine whether HC-use does exert a protective effect against BV.

A number of important limitations were present in this meta-analysis. First, the meta-analysis was limited to published studies, which could overestimate the overall estimates if there has been publication bias resulting from the tendency to publish and present only statistically significant findings. We only searched studies which were published in English, which may limit the generalizability of our findings; however included studies represented women in all continents and from diverse ethnicities. Importantly, no evidence of publication bias was seen in either funnel plot or in the Eggers test for bias, and in a number of studies where raw data was presented, we included derived associations that were not mentioned in the manuscript. A potential limitation is the inclusion of clinical trials and quite specific sub-populations. While this may have also contributed to bias, we conducted sensitivity analyses and showed that their inclusion did not significantly affect the overall effect size. Although we included adjusted estimates where possible, unmeasured confounding may have contributed to the pooled estimates i.e. there may have been other unmeasured biases contributing to women’s choice of HC, which was not adjusted for in analyses and may have resulted in an overestimation of the effect. One of the strengths of this meta-analysis was that it included highly diverse studies from many different geographical locations, and women with diverse risks from various recruitment settings, but there were more women recruited from sexual/reproductive health services compared to broader population-based studies. This may somewhat limit the generalizability of the findings, and could be a source of bias, however, as the negative association is robust across these heterogeneous studies, this indicates the impact of selection bias is minimal. A significant proportion of studies did not specify type of HC-use. This may have disproportionately affected the associations between POC-use and BV outcomes in for instance African settings, and combined HC-use in developed nation settings, where each of these methods is, respectively, more commonly used. Overall, however, this is likely to have limited impact on the pooled estimates. Finally, the control groups varied between studies and often contained IUD-users, users of other HC-types and condom users. IUD-use, which predominantly reflected non-hormonal IUDs, has importantly been associated with increased risk of BV [75]. For this reason, we excluded any studies that exclusively had IUD-users as the control population as this would lead to an overestimation of the effect, but importantly, for the majority of other studies, IUD-users represented only a minority of the control population. It is reasonable to assume that many HC-users may use condoms less consistently than non-HC users. However, a previous meta-analysis has shown that condom use is associated with a 20% reduced risk for BV [76], and therefore the inclusion of a greater proportion of consistent condom users in control populations, is more likely to underestimate, rather than overestimate, an observed protective effect of HC against BV. Importantly, we included ratios that had been adjusted for condom use when provided. The most striking observation from these data is that the negative association between HC-use and BV was robust and consistent when stratified by HC-type and across the three outcome measures.

In conclusion, this meta-analysis demonstrates a negative association between HC-use and the risk of BV, and raises the tantalizing potential role of exogenous steroid hormones in influencing the vaginal environment in a protective manner against the development of BV. With over 50% of women experiencing BV recurrence following first-line antibiotic therapies, and no significant improvement in the management of BV in the last 20 years, identifying potential modifiable sexual and contraceptive practices that influence susceptibility to infection and recurrence are integral to progressing prevention and management approaches for this important and common genital tract infection. Crucially, there are no data from RCTs evaluating a hormonal intervention, and the mechanism(s) by which hormonal contraception may exert a protective effect against BV requires further investigation.

## Supporting Information

Figure S1 **Prevalence of bacterial vaginosis (BV) in prevalence studies, stratified by geographical location.** Key: nNUM = population size, CI = confidence interval
(TIF)

Figure S2 **Prevalence of bacterial vaginosis (BV) in prevalence studies, stratified by BV diagnostic method (Amsel/modified Amsel method compared to Nugent method).** Three studies used other methods (Ison-Hay, Spiegal and one study that used both Amsel and Nugent methods) and were not included in this figure. Key: nNUM = population size
(TIF)

Figure S3 **Prevalence of bacterial vaginosis (BV) in prevalence studies, stratified by recruitment setting.** Key: nNUM = population size, SRHS = sexual or reproductive health service, GCHS = general community healthcare service, SWS = sex worker service, POP = population based
(TIF)

Table S1 **Sensitivity analyses of all prevalent BV studies included in the meta-analysis, stratified by hormonal contraceptive type used.**
(DOCX)

Table S2 **Sensitivity analyses of all incident BV studies included in the meta-analysis, stratified by hormonal contraceptive type used.**
(DOCX)

Table S3 **Sensitivity analyses of all studies included in the meta-analysis, stratified by BV outcome measure (prevalent, incident, recurrent BV).**
(DOCX)
