# Supplementary figures and images for: Hormonal Contraception Is Associated with a Reduced Risk of Bacterial Vaginosis: A Systematic Review and Meta-Analysis
Source: PLoS One. 2013 Sep 4;8(9):e73055. doi: 10.1371/journal.pone.0073055 (PMC3762860; doi:10.1371/journal.pone.0073055)

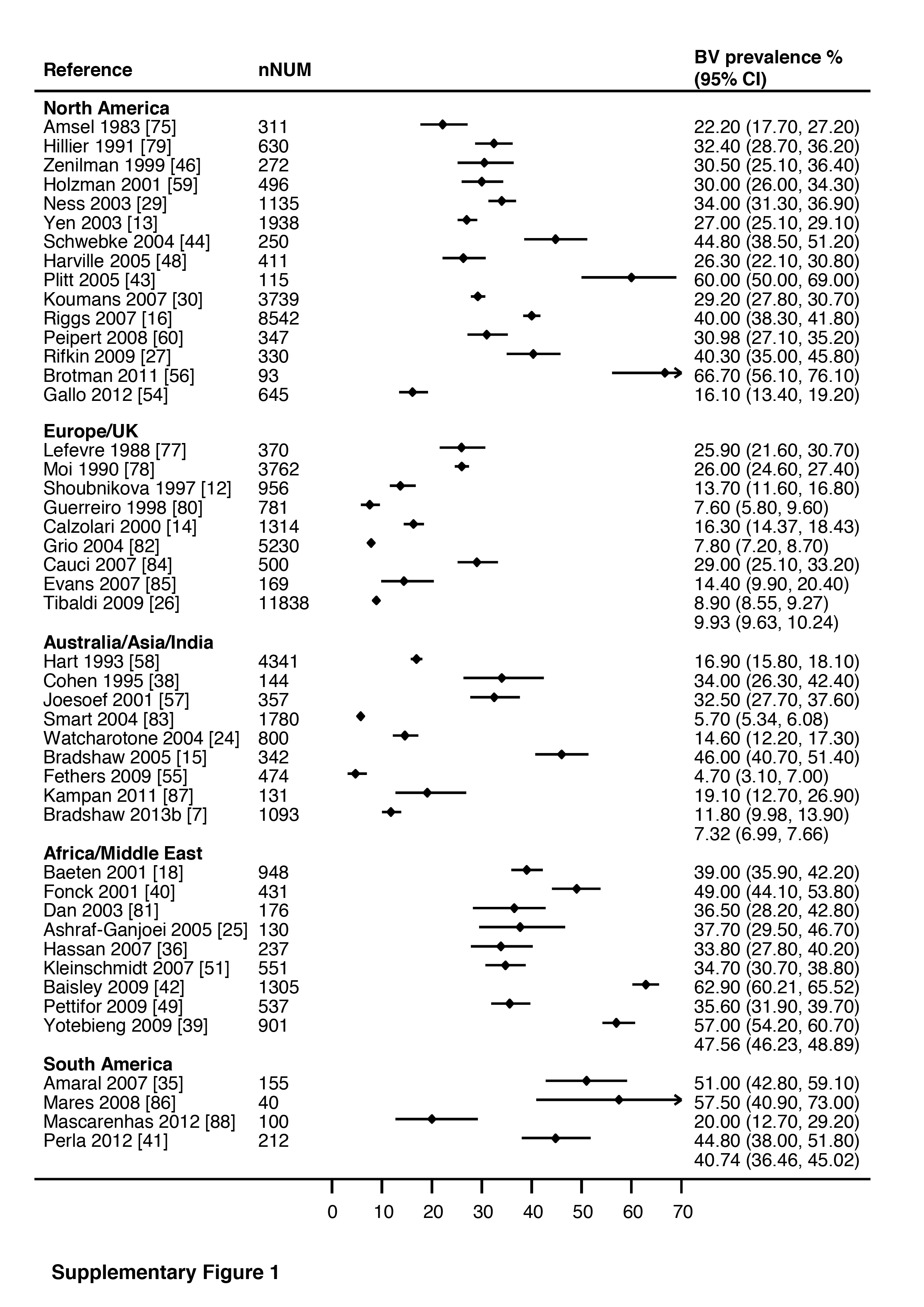

Supplement: Figure S1 — Prevalence of bacterial vaginosis (BV) in prevalence studies, stratified by geographical location. Key: nNUM = population size, CI = confidence interval (TIF) [file pone.0073055.s001.tif]

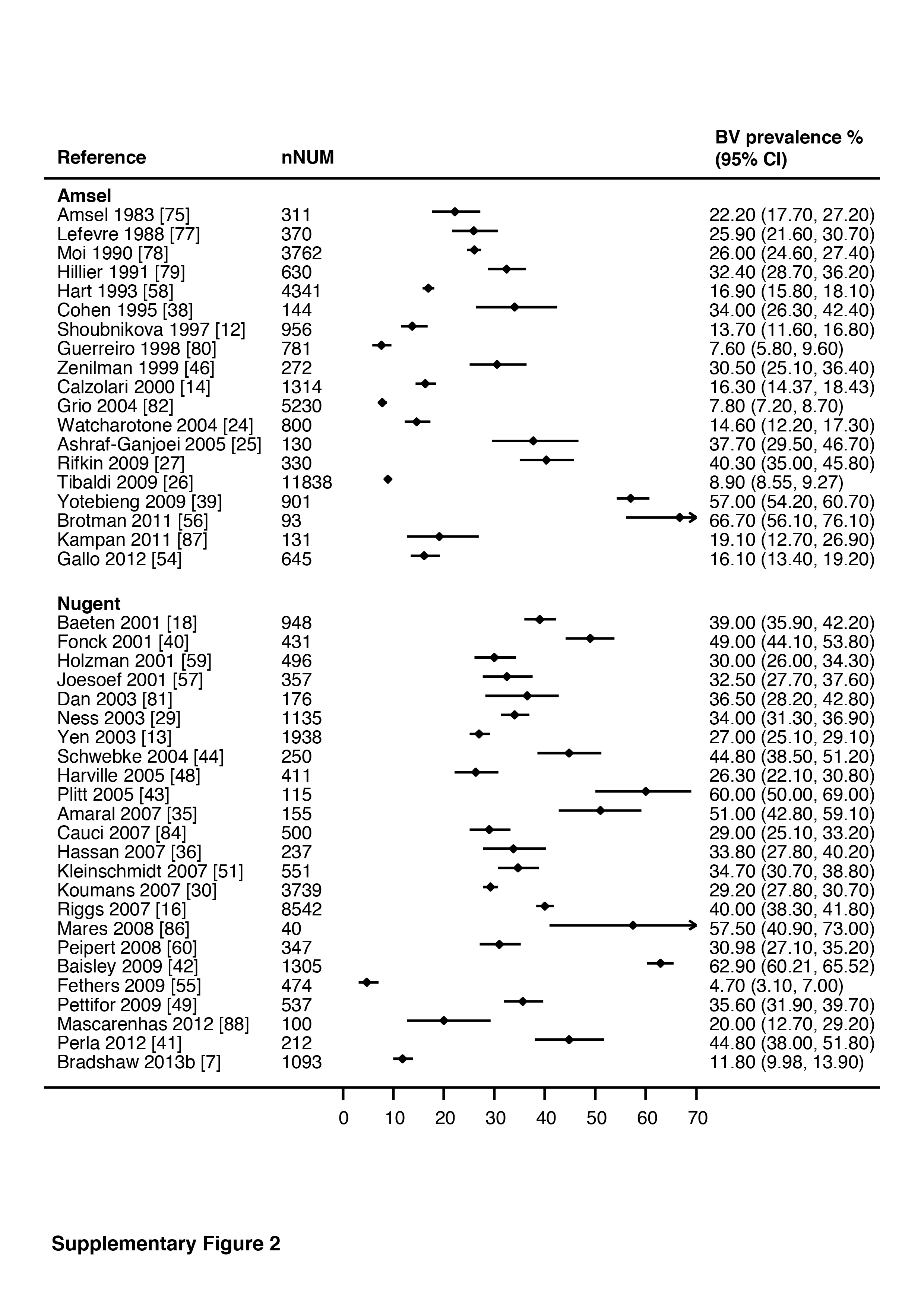

Supplement: Figure S2 — Prevalence of bacterial vaginosis (BV) in prevalence studies, stratified by BV diagnostic method (Amsel/modified Amsel method compared to Nugent method). Three studies used other methods (Ison-Hay, Spiegal and one study that used both Amsel and Nugent methods) and were not included in this figure. Key: nNUM = population size (TIF) [file pone.0073055.s002.tif]

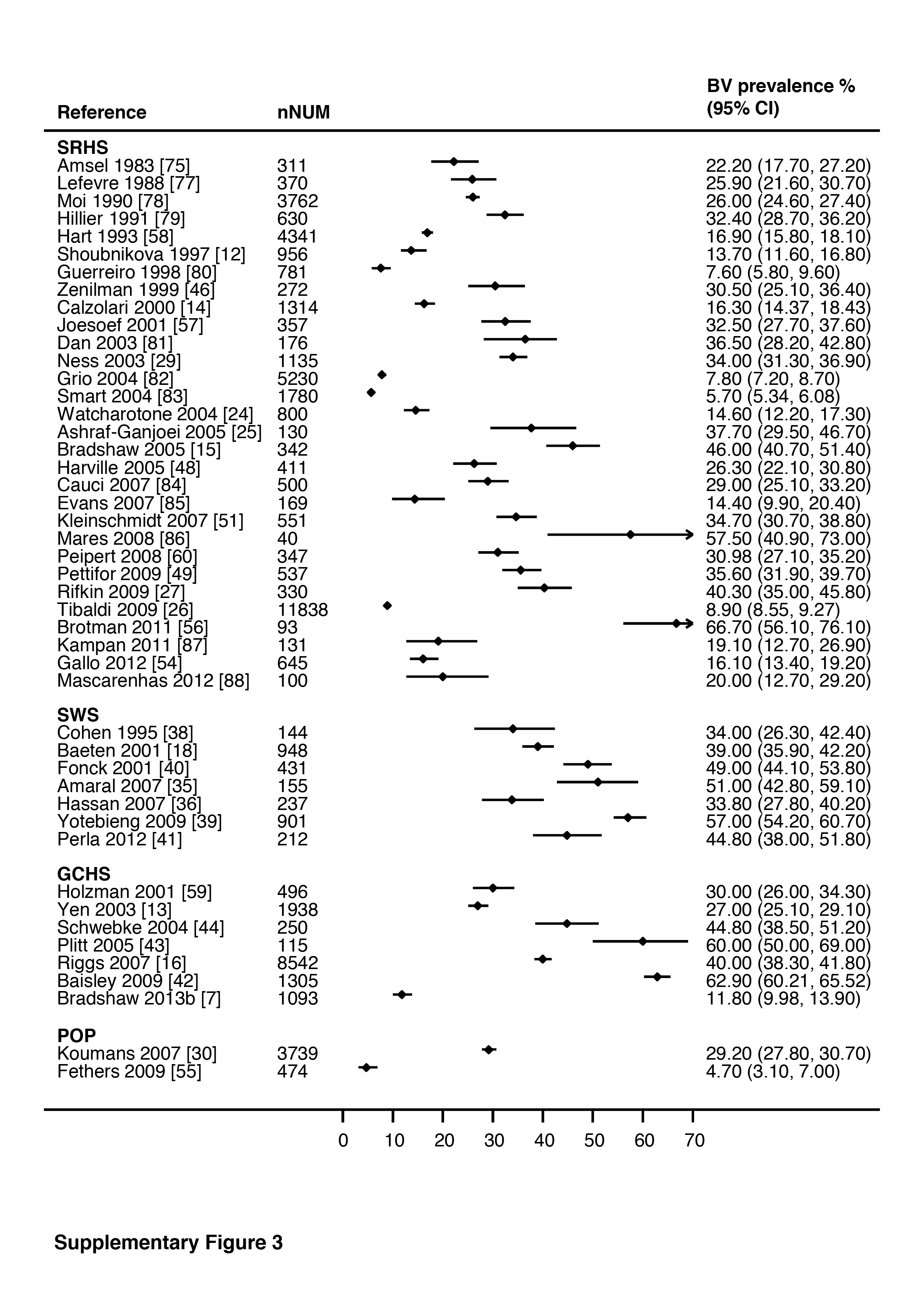

Supplement: Figure S3 — Prevalence of bacterial vaginosis (BV) in prevalence studies, stratified by recruitment setting. Key: nNUM = population size, SRHS = sexual or reproductive health service, GCHS = general community healthcare service, SWS = sex worker service, POP = population based (TIF) [file pone.0073055.s003.tif]
