# Supplementary material for: Hormonal Contraception Is Associated with a Reduced Risk of Bacterial Vaginosis: A Systematic Review and Meta-Analysis
Source: PLoS One. 2013 Sep 4;8(9):e73055. doi: 10.1371/journal.pone.0073055 (PMC3762860; doi:10.1371/journal.pone.0073055)
Supplement: Table S1 — Sensitivity analyses of all prevalent BV studies included in the meta-analysis, stratified by hormonal contraceptive type used. (DOCX) [file pone.0073055.s004.docx]

**Table S1:** Sensitivity analyses of prevalent BV studies included in the meta-analysis, stratified by hormonal contraceptive type used.

|  | Overall pooled ES | Combined HC-use pooled ES | POC HC-use pooled ES | Unspecified HC-use pooled ES |
| --- | --- | --- | --- | --- |
|  | (95% CI) | (95% CI) | (95% CI) | (95% CI) |
| All prevalent BV studies | 0.68 (0.64-0.73)  n=47 | 0.72 (0.66-0.78)  n=24 | 0.72 (0.57-0.90)  n=6 | 0.64 (0.55-0.74)  n=17 |
| *Sensitivity analyses excluding sub-groups^a^* | | | | |
| excluding RCTs | 0.69 (0.64-0.73) n=41 | 0.72 (0.67-0.77)  n=22 | 0.72 (0.54-0.96)  n=5 | 0.64 (0.54-0.75)  n=14 |
| excluding SW | 0.69 (0.65-0.74)  n=40 | 0.72 (0.67-0.77)  n=21 | 0.70 (0.51-0.96)  n=4 | 0.66 (0.57-0.77)  n=15 |
| excluding all specific populations^b^ | 0.69 (0.64-0.74)  n=38 | 0.72 (0.67-0.77)  n=21 | 0.70 (0.51-0.96)  n=4 | 0.64 (0.53-0.76)  n=13 |
| excludes women not using contraceptives in control groups NC/TL | 0.69 (0.64-0.74)  n=44 | 0.72 (0.66-0.78)  n=23 | 0.74 (0.60-0.91)  n=5 | 0.63 (0.54-0.73)  n=16 |
| excluding studies which group intermediate NS with BV | 0.69 (0.64-0.74)  n=45 | 0.72 (0.66-0.78)  n=24 | 0.72 (0.57-0.90)  n=6 | 0.64 (0.54-0.76)  n=15 |
| *Examples of individual sensitivity analyses^ac^* | | | | |
| excluding lowest  combined HC-use PE | 0.69 (0.64-0.74) | 0.73 (0.68-0.79) |  |  |
| excluding highest  combined HC-use PE | 0.69 (0.64-0.74) | 0.72 (0.67-0.77) |  |  |
| excluding lowest  POC HC-use PE | 0.69 (0.64-0.74) |  | 0.74 (0.60-0.91) |  |
| excluding highest POC HC-use PE | 0.68 (0.63-0.74) |  | 0.71 (0.55-0.91) |  |
| excluding lowest unspecified HC-use PE | 0.70 (0.66-0.75) |  |  | 0.67 (0.60-0.74) |
| excluding highest unspecified HC-use PE | 0.68 (0.63-0.74) |  |  | 0.63 (0.55-0.73) |

^a^all effect size estimates are random-effects; ^b^all participants are sex workers (SW), all participants have herpes simplex virus 2 (HSV2) (Baisley 2009), or all participants douche (Schwebke 2004); ^c^individual analyses were removed one by one with no significant change to the overall effect size estimates, examples are shown;

Key: ES= effect size; HC=hormonal contraception, POC=progesterone only containing; RCT=studies utilising data from women screened or enrolled in randomised controlled trial; n= number of associations contributing to the overall ES; SW=all participants are sex workers; NC/TL=not using any contraception/tubal ligation; PE=point estimate
