# Supplementary material for: Hormonal Contraception Is Associated with a Reduced Risk of Bacterial Vaginosis: A Systematic Review and Meta-Analysis
Source: PLoS One. 2013 Sep 4;8(9):e73055. doi: 10.1371/journal.pone.0073055 (PMC3762860; doi:10.1371/journal.pone.0073055)
Supplement: Table S2 — Sensitivity analyses of all incident BV studies included in the meta-analysis, stratified by hormonal contraceptive type used. (DOCX) [file pone.0073055.s005.docx]

**Table S2:** Sensitivity analyses of incident BV studies included in the meta-analysis, stratified by hormonal contraceptive type used.

|  | Overall pooled ES | Combined HC-use pooled ES | POC HC-use pooled ES | Unspecified HC-use pooled ES |
| --- | --- | --- | --- | --- |
|  | (95% CI) | (95% CI) | (95% CI) | (95% CI) |
| All incident BV studies | 0.82 (0.72-0.92) n=14 | 0.84 (0.73-0.98) n=7 | 0.81 (0.65-0.99) n=6 | 0.49 (0.17-1.40)^c^ n=1 |
| *Sensitivity analyses excluding sub-groups^a^* | | | | |
| excluding RCTs | 0.85 (0.75-0.96) n=12 | 0.84 (0.71-1.00)  n=6 | 0.87 (0.72-1.05) n=5 |  |
| excluding SW | 0.87 (0.76-0.99)  n=11 | 0.85 (0.71-1.02)  n=6 | 0.91 (0.74-1.13)  n=4 |  |
| excluding specific populations^b^ | 0.87 (0.76-0.99)  n=11 | 0.85 (0.71-1.02)  n=6 | 0.91 (0.74-1.13)  n=4 |  |
| excludes women not using contraceptives in control groups NC/TL | 0.85 (0.74-0.99)  n=9 | 0.86 (0.69-1.07)  n=5 | 0.86 (0.69-1.07)  n=3 |  |
| *Examples of individual sensitivity analyses^ad^* | | | | |
| excluding lowest  combined HC-use PE | 0.83 (0.74-0.94) | 0.88 (0.77-1.01) |  |  |
| excluding highest  combined HC-use PE | 0.79 (0.71-0.89) | 0.80 (0.72-0.90) |  |  |
| excluding lowest  POC HC-use PE | 0.85 (0.76-0.95) |  | 0.87 (0.72-1.05) |  |
| excluding highest POC HC-use PE | 0.80 (0.71-0.90) |  | 0.76 (0.62-0.93) |  |
| excluding unspecified HC-use PE | 0.82 (0.73-0.93) |  |  | none |

^a^all effect size estimates are random-effects; ^b^all participants are sex workers (SW), all participants have HSV2+ (Baisley 2009), or all participants douche (Schwebke 2004); ^c^one study (Schwebke, 2005) contributes unspecified HC-use to pooled estimates of the association of HC-use on BV incidence but no pooled estimate available for unspecified HC-use and incident BV; ^d^individual analyses were removed one by one with no significant change to the overall effect size estimates, examples are shown.

Key: ES= effect size; HC=hormonal contraception, POC=progesterone only containing; RCT=studies utilising data from women screened or enrolled in randomised controlled trial; n= number of associations contributing to the overall ES; SW=all participants are sex workers; NC/TL=not using any contraception/tubal ligation; PE=point estimate
