# Supplementary material for: Hormonal Contraception Is Associated with a Reduced Risk of Bacterial Vaginosis: A Systematic Review and Meta-Analysis
Source: PLoS One. 2013 Sep 4;8(9):e73055. doi: 10.1371/journal.pone.0073055 (PMC3762860; doi:10.1371/journal.pone.0073055)
Supplement: Table S3 — Sensitivity analyses of all studies included in the meta-analysis, stratified by BV outcome measure (prevalent, incident, recurrent BV). (DOCX) [file pone.0073055.s006.docx]

**Table S3:** Sensitivity analyses of all studies included in the meta-analysis, stratified by BV outcome measure (prevalent, incident, recurrent BV).

|  | Overall  pooled ES | Prevalent BV pooled ES | Incident BV pooled ES | Recurrent BV pooled ES |
| --- | --- | --- | --- | --- |
|  | (95% CI) | (95% CI) | (95% CI) | (95% CI) |
| All studies^a^ | 0.79 (0.75-0.82)  n=66 | 0.79 (0.75-0.83)  n=47 | 0.82 (0.73-0.92)  n=14 | 0.69 (0.52-0.91)  n=5 |
| *Sensitivity analyses excluding sub-groups^b^* | | | | |
| excluding RCTs | 0.78 (0.74-0.82)  n=56 | 0.77 (0.73-0.81)  n=41 | 0.85 (0.75-0.96)  n=12 | 0.65 (0.53-0.80)  n=3 |
| excluding SW | 0.79 (0.75-0.83)  n=56 | 0.78 (0.74-0.82)  n=40 | 0.87 (0.76-0.99)  n=11 | 0.69 (0.52-0.91)  n=5 |
| excluding specific populations^c^ | 0.78 (0.74-0.82)  n=54 | 0.77 (0.73-0.82)  n=38 | 0.87 (0.76-0.99)  n=11 | 0.69 (0.52-0.91)  n=5 |
| excludes women not using contraceptives in control groups NC/TL | 0.78 (0.74-0.82)  n=58 | 0.78 (0.73-0.82)  n=44 | 0.85 (0.74-0.99)  n=9 | 0.69 (0.52-0.91)  n=5 |
| excluding studies which group intermediate NS with BV | 0.78 (0.74-0.82)  n=64 | 0.78 (0.73-0.82)  n=45 | 0.82 (0.73-0.92)  n=14 | 0.69 (0.52-0.91)  n=5 |
| *Examples of individual sensitivity analyses^bd^* | | | | |
| Excluding lowest prevalence PE | 0.79 (0.75-0.82) | 0.79 (0.75-0.83) |  |  |
| Excluding highest prevalence PE | 0.77 (0.74-0.81) | 0.77 (0.73-0.81) |  |  |
| Excluding lowest incidence PE | 0.78 (0.74-0.82) |  | 0.82 (0.73-0.93) |  |
| Excluding highest incidence PE | 0.78 (0.74-0.82) |  | 0.80 (0.71-0.90) |  |
| Excluding lowest recurrence PE | 0.78 (0.74-0.82) |  |  | 0.72 (0.53-0.97) |
| Excluding highest recurrence PE | 0.78 (0.74-0.82) |  |  | 0.63 (0.52-0.75) |

^a^all odds ratios were converted to risk ratios for calculation of the association of any BV with use of hormonal contraception; ^b^all effect size estimates are random-effects; ^c^all participants are sex workers (SW), all participants have HSV2+ (Baisley 2009), or all participants douche (Schwebke 2004); ^d^individual analyses were removed one by one with no significant change to the overall effect size estimates, examples are shown.

Key: ES= effect size; HC=hormonal contraception, POC=progesterone only containing; RCT=studies utilising data from women screened or enrolled in randomised controlled trial; n=number of associations contributing to the overall ES; SW=all participants are sex workers; NC/TL=not using any contraception/tubal ligation; PE=point estimate
